# How Facial Expressions of Emotion Affect Distance Perception

**DOI:** 10.3389/fpsyg.2015.01825

**Published:** 2015-11-24

**Authors:** Nam-Gyoon Kim, Heejung Son

**Affiliations:** Department of Psychology, Keimyung University, Daegu, Korea

**Keywords:** emotional facial expressions, behavioral intention, approach-avoidance behavior, distance perception, gender difference

## Abstract

Facial expressions of emotion are thought to convey expressers’ behavioral intentions, thus priming observers’ approach and avoidance tendencies appropriately. The present study examined whether detecting expressions of behavioral intent influences perceivers’ estimation of the expresser’s distance from them. Eighteen undergraduates (nine male and nine female) participated in the study. Six facial expressions were chosen on the basis of degree of threat—anger, hate (threatening expressions), shame, surprise (neutral expressions), pleasure, and joy (safe expressions). Each facial expression was presented on a tablet PC held by an assistant covered by a black drape who stood 1, 2, or 3 m away from participants. Participants performed a visual matching task to report the perceived distance. Results showed that facial expression influenced distance estimation, with faces exhibiting threatening or safe expressions judged closer than those showing neutral expressions. Females’ judgments were more likely to be influenced; but these influences largely disappeared beyond the 2 m distance. These results suggest that facial expressions of emotion (particularly threatening or safe emotions) influence others’ (especially females’) distance estimations but only within close proximity.

Face is the primary channel through which emotions are expressed (Ekman, 1965). Using facial expressions, individuals make their feelings known to others. By decoding facial signals, observers can understand an expresser’s emotion. Moreover, emotions have been directly linked to motivational tendencies (Frijda, 1986, 1988; Lang et al., 1990; Izard, 1994; Davidson, 1998; Keltner and Haidt, 2001; Carver, 2006; Reis and Gray, 2009; Lowe and Ziemke, 2011) and may enable perceivers to anticipate the expresser’s behavioral intentions (Fridlund, 1994; Horstmann, 2003; Seidel et al., 2010). Anticipating behavioral intentions can trigger appropriate approach or avoidance (withdrawal) responses by the perceiver. That is, the perceiver is primed to approach expressers whose facial expressions are positive (e.g., joy) or avoid those whose expressions are negative (e.g., anger). Thus, detecting another’s behavioral intention through their facial expressions plays a vital role in social interaction.

During the last decade, research on behavioral intentions in emotion has attracted enormous interest (see Eder et al., 2013). The research has largely focused on the physiological changes experienced, or the corresponding approach-avoidance responses executed, by the perceiver (Adams and Kleck, 2003, 2005; Adams et al., 2006; Nelson et al., 2013; see also Eder et al., 2013). Research on the perceptual processes involved in detecting the behavioral intentions conveyed by emotional facial expressions has garnered much less interest, although some have begun to explore issues relevant to detecting information about emotion from facial expressions (Adams and Kleck, 2003, 2005; Adams et al., 2006; Nelson et al., 2013). For example, Adams and Kleck (2003, 2005) demonstrated that the gaze direction of the face displaying an emotion enhances the identification of the displayed emotion if gaze direction coincides with the underlying behavioral tendency associated with the emotion, that is, direct gaze facilitating the identification of facial emotions associated with approach (e.g., anger and joy) but averted gaze facilitating the identification of facial emotions associated with withdrawal (e.g., fear and sadness).

We examined how perception of behavioral intentions from facial expressions of emotion affects estimates of distances to the person expressing the emotion. For example, imagine a woman facing an angry man. Detecting the anger in his facial expression, the woman’s emotional system activates an avoidance tendency that prepares her to flee or otherwise protect herself in case he attacks. Knowing how far away the man is from her is critical to her ability to escape harm.

In such a threatening circumstance, would a woman be able to perceive the distance to him accurately? That is, would the perceived intent of the potential aggressor influence her capacity to estimate distance accurately? In fact, there is reason to suspect that it might. Teachman et al. (2008) conducted a study in which participants estimated the vertical height from a two-story, 26-foot balcony. In this study, the participants were divided into high and low acrophobia groups based on their symptoms. Although both groups overestimated vertical heights, the degree of overestimation was exaggerated in the high fear group. The result was construed as evidence for acrophobia biasing perceptual judgments of height.

The present study differs from Teachman et al.’s (2008) study on several grounds. First, in the present study, participants’ affect states were induced by photos of others’ depictions of emotion through facial expressions. The behavioral intentions underlying the emotions demonstrated facially are likely to be the cause of any perceptual bias in distance judgment. Teachman et al. (2008) manipulated a single emotional state (fear of heights); but the present study employed several affective stimuli, each varying as to degree of threat, to elicit different emotional responses in participants. In the Teachman et al. (2008) study, distance estimations were compared with the actual height of a 26-foot balcony (a fairly large space). The stimuli used in the present study were confined within a close (3 m radii) social space to facilitate their mediating roles in social interaction. It is generally believed that women are superior to men in experiencing and expressing emotions (Hall, 1978; Eisenberg and Lennon, 1983; Barrett et al., 2000; Hall et al., 2000; see Kret and De Gelder, 2012, for a review). However, empirical evidence for women’s advantage in the recognition of emotional facial expressions has been inconclusive (Hampson et al., 2006). Thus, in this study, we also examined whether gender affects distance estimation over and above the behavioral intentions detected from emotional facial expressions. Marsh et al. (2005) demonstrated faster responses to female faces, but Rotteveel and Phaf (2004) found the opposite pattern. Hoping to clarify these conflicting findings, we also examined the influence of the gender of the actor producing the emotion on distance estimation.

## Materials and Methods

### Participants

Eighteen undergraduates (nine male and nine female) from Keimyung University volunteered for the study for partial course credit. All participants had normal or corrected-to-normal vision.

### Ethics Statement

The study was approved by the ethics committee at Keimyung University. After complete description of the study to the participants, written informed consent was obtained in accordance with the Declaration of Helsinki.

### Materials

Twelve facial photographs of two actors (one male and one female) displaying six emotional expressions (pleasure, joy, surprise, shame, hate, and anger) were employed in the study. These photographs, standard VGA images of 480 H × 640 V pixels, were selected from the Face Database developed by Yonsei University Center for Cognitive Science (1998). This database is comprised of six sets of 83 facial photographs with each set including 22 “pure” expressions posed by one of six Korean actors (four amateur and two professional; three male and three female). Based on picture quality, four sets by four amateur actors were excluded. From the remaining two sets, photographs depicting 22 “pure” expressions from each set were rated by 10 judges (five male and five female volunteers, all undergraduates from Keimyung University) in terms of degree of threat using a 7-point rating scale with one for safest and seven for most threatening. Based on the average rating of each photograph, two facial expressions with the lowest scores (safe expressions: pleasure and joy), two expressions with mid-range scores (neutral expressions: surprise and shame), and two expressions with the highest scores (threatening expressions: anger and hate) were chosen for the experiment. The average ratings for the six expressions were pleasure (*M* = 1.8, SD = 0.86), joy (*M* = 2.2, SD = 1.0), surprise (*M* = 4.1, SD = 0.90), shame (*M* = 4.2, SD = 1.10), hate (*M* = 5.1, SD = 0.75), and anger (*M* = 5.4, SD = 1.06), respectively. Thus, the photos used in the experiment comprised six standard photos of a male actor and six of a female actor displaying the same six “pure” expressions of emotion.

### Design

Four variables were controlled in the experiment: participant gender, actor gender, emotional expression, and distance. Thus, the experiment utilized a 2 (Participant Gender) × 2 (Actor Gender) × 6 (Emotion: pleasure, joy, shame, surprise, hate, and anger) × 3 (Distance: 1, 2, 3 m) mixed-design for a total of 36 trials. Participant gender was controlled between-subjects, and the other three variables were controlled within-subjects. All trials were randomized for each participant.

### Procedure

**FIGURE 1:**
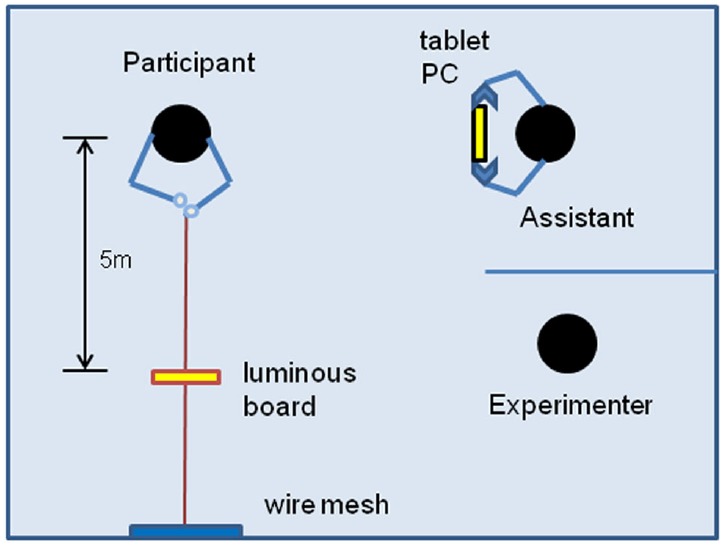
**The layout of the room in which the experiment took place.** See text for details.

Following Teachman et al. (2008), participants performed a visual matching task to report the perceived distance of each face from them. According to Teachman et al. (2008), a visual matching task is an effective measure of perceptual effects because it is less susceptible to cognitive biases than measures based on verbal report or memory. The visual matching experiment was conducted in a 7 m × 7 m room in which individual participants stood in one corner facing a wall 6 m in front of them (see Figure 1). A 26 cm × 70 cm wire mesh with a 3.5 cm aperture was hung on the wall at height of 1.9 m. A fishing line was wrapped around a mesh at the participant’s eye level, and the ends of the fishing line then were tied together. Attached to the fishing line was an 11 cm × 11 cm fluorescent panel that participants could move toward or away from themselves by pulling the upper or lower end of the fishing line, respectively. The panel consisted simply of a cardboard square covered by yellow fluorescent tape.

Stimuli were presented on a 10.1 inch tablet PC (Samsung Galaxy Tab Pro 10.1, Samsung Electronics, Suwon, Korea) with a pixel resolution of 1280 H × 800 V. An assistant wearing a black drape and black gloves, and standing 90° to the left of the participant, held the 470 g tablet PC so that its center aligned with the participant’s eye level and the height of the fishing line. To complete the task, participants first turned their heads 90° to the left to view the face on the PC and then looked straight ahead to move the fluorescent panel to the perceived distance by manipulating the fishing line. The experiment was conducted in a dark room to exclude pictorial cues (e.g., texture gradients, shading, and linear perspective) that might facilitate distance estimation. Consequently, the display panel of the PC and the fluorescent board were the only significant sources of light. Nevertheless, other potential sources of information for distance estimation (e.g., accommodation, convergence, binocular disparity, and motion parallax) were available.

Participants were tested individually. Before the experiment, participants completed a consent form and the experimenter measured their eye heights. After explaining the task, the experimenter encouraged participants to manipulate the visual matching device and familiarize themselves with its use. When participants reported feeling comfortable manipulating the device, the experimental trials began.

While the experimenter and the assistant were preparing for each trial, participants were asked to close their eyes and maintain their positions. The experimenter then positioned the fluorescent panel 5 m from the participant, and the assistant moved to the distance set for that trial. Once the fluorescent panel and assistant were in position, the experimenter also moved behind a divider to remain invisible (see Figure 1). The experimenter then asked participants to open their eyes and begin the task. Participants were allowed to turn to view the stimulus and then readjust the fluorescent panel repeatedly until they were completely satisfied with their distance estimates. At that point, the experimenter, with the help of the assistant, measured the distance between the fluorescent panel and the outer canthus of the participant’s left eye. It should be noted that participants faced the wall to manipulate the reporting device to report the estimated distance while turning their heads to the left to view the stimulus resulting in the misalignment of the head/eye-centered frame of reference with the body-centered frame of reference. Whether this misalignment influenced accurate registration of the distances to the stimuli is unknown. However, any effect would have been the same for all conditions in the present study. Holway and Boring (1941) employed a similar setup in which participants sat on a chair facing the standard stimulus and then adjusted the comparison stimulus lying to their right.

The same procedure was repeated for each trial. No feedback was provided during the experiment. The entire experiment lasted about 50 min.

## Results

Participants’ responses were converted to constant errors for analysis. Constant error represents participants’ average response error and the directional bias of these errors. Constant error values were entered into a mixed-design analysis of variance (ANOVA) for analysis with participant gender, actor gender, facial emotion and distance as independent variables. When the ANOVA sphericity assumption was violated (Mauchly’s test, *p* < 0.05), we used the Greenhouse–Geisser correction (Winer, 1971). The fractional degrees of freedom indicate this correction. Prior to the ANOVA, we also performed Shapiro-Wilk tests on dependent variables to ensure that the assumption of normality was valid. All results were insignificant (*p* > 0.05), confirming the normal distribution.

The ANOVA confirmed main effects of distance, *F*(1.4,22.9) = 4.62, *p* < 0.05, ${\text{η}}_{p}^{2}$ = 0.22, and emotion, *F*(5,80) = 5.63, *p* < 0.0001, ${\text{η}}_{p}^{2}$ = 0.26. Neither participant nor actor gender reached statistical significance, *F* < 1, *ns*. A Tukey *post hoc* test confirmed performance differences between the 1 m condition and the 3 m condition at the 0.05 level. A Tukey test also confirmed that the perceived distances to the two neutral expressions were different from those to the two threatening expressions, at the 0.0001 level.

**FIGURE 2:**
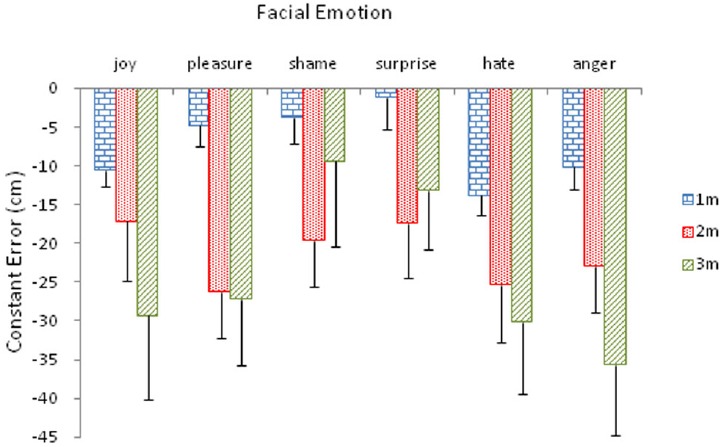
**Mean constant error (with standard error bars) as a function of facial expressions of emotion for the three distance conditions**.

These differences were further qualified by the Distance × Emotion interaction, *F*(10,160) = 2.10, *p* < 0.05, ${\text{η}}_{p}^{2}$ = 0.12 (Figure 2). A simple effects analysis confirmed that the effect of distance was significant for pleasure, *F*(2,15) = 8.89, *p* < 0.01, shame, *F*(2,15) = 8.47, *p* < 0.01, surprise, *F*(2,15) = 4.58, *p* < 0.05, and anger, *F*(2,15) = 4.73, *p* < 0.05; whereas the effect of facial emotion was significant in the 1 m condition, *F*(5,12) = 4.69, *p* < 0.05, marginally significant in the 2 m condition, *F*(5,12) = 3.10, *p* = 0.05, but insignificant in the 3 m condition, *F*(5,12) = 2.48, *p* = 0.09.

To examine the source of this interaction, pairwise comparisons among distance conditions for each emotion and then among emotion conditions for each distance were performed individually. With respect to the distance effect, performance in the 1 m and 2 m conditions differed from each other for pleasure (*p* < 0.01), shame (*p* < 0.01), surprise (*p* < 0.01) and anger (*p* < 0.05); whereas performance in the 1 m and 3 m conditions differed from each other (*p* < 0.01). With respect to the emotion effect, in the 1 m condition, shame and surprise each differed from joy and anger at the 0.05 level. Shame differed from hate (*p* < 0.001) and surprise differed from hate (*p* < 0.01). In addition, pleasure differed from hate (*p* < 0.05); and hate and anger differed from each other (*p* < 0.05). In the 2 m condition, there were no significant differences among means.

**FIGURE 3:**
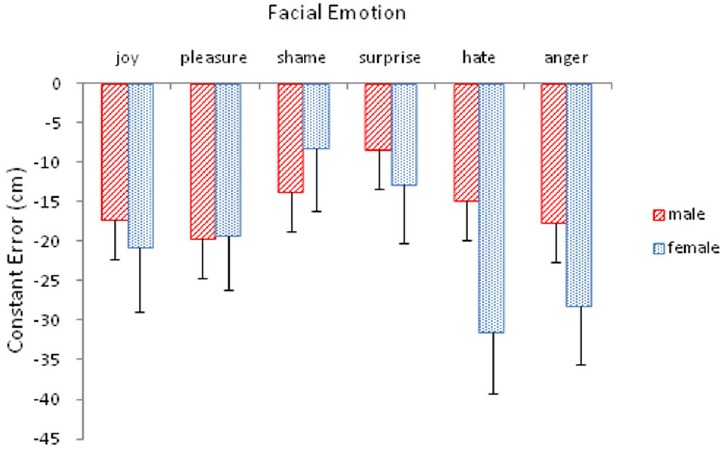
**Mean constant error (with standard error bars) as a function of facial expressions of emotion for male and female participants**.

Emotion also interacted with participant gender, *F*(5,80) = 2.72, *p* < 0.05, ${\text{η}}_{p}^{2}$ = 0.15 (Figure 3). A simple effects analysis revealed that this interaction arose from the significant effect of participant gender, particularly, female participants, *F*(5,12) = 5.30, *p* < 0.01. As shown in Figure 2, the extent of underestimation by female participants was particularly pronounced for hate and anger, the two threatening expressions. For female participants, pairwise comparisons among emotion types showed that hate differed significantly from joy (*p* < 0.05), pleasure (*p* < 0.05), shame (*p* < 0.001), and surprise (*p* < 0.01); and anger differed significantly from pleasure (*p* < 0.05), shame (*p* < 0.01), and surprise (*p* < 0.05). In addition, shame also differed from joy (*p* < 0.01) and pleasure (*p* < 0.05).

Irrespective of emotions displayed, it appears that faces were perceived as closer than they actually were (Figures 2 and 3). Indeed, the mean constant errors (SD) for the six facial emotions of joy, pleasure, shame, surprise, hate, and anger were –19.04 (23.66), –19.45 (19.98), –10.96 (22.99), –10.60 (21.66), –23.14 (24.44) and –22.94 (22.02) cm, respectively. However, one sample *t*-tests revealed that the two safe expressions [*t*(17) = –3.41, *p* = 0.003, for joy; *t*(17) = –4.13, *p* = 0.0001, for pleasure] and two threatening expressions [*t*(17) = –4.02, *p* = 0.001, for hate; *t*(17) = –4.42, *p* < 0.0001, for anger] were underestimated but not the two neutral emotions [*t*(17) = –2.02, *p* = 0.059 for shame; *t*(17) = –2.08, *p* = 0.053 for surprise].

The preceding analysis was performed on the mean constant errors averaged across the three distance conditions. To examine how each of these emotional expressions was perceived under each distance condition, responses were divided along each distance condition and a separate one sample *t*-test was performed on each facial emotion (see Figure 2). In the 1 m condition, joy, *t*(17) = –4.72, *p* < 0.0001, hate, *t*(17) = –4.28, *p* < 0.01, and anger, *t*(17) = –2.96, *p* < 0.01, were underestimated. In the 2 m condition, however, all emotions were underestimated, at least at the 0.05 level. In the 3 m condition, on the other hand, in addition to the three emotions found significant in the 1 m condition [joy, *t*(17) = –2.76, *p* < 0.05, hate, *t*(17) = –3.28, *p* < 0.01, and anger, *t*(17) = –4.03, *p* < 0.01], pleasure, *t*(17) = –3.21, *p* < 0.01, was also found to be underestimated.

## Discussion

Facial expressions of emotion play a vital role in human social interaction. Indeed, numerous studies have demonstrated that observers can recognize from facial expressions, not only the emotional state, but also the behavioral intentions or action demands of the expresser (Fridlund, 1994; Adams and Kleck, 2003, 2005; Horstmann, 2003; Adams et al., 2006; Seidel et al., 2010), allowing observers to respond appropriately (see Phaf et al., 2014, for review). We explored whether the detected behavioral intentions influence one’s capacity to perceive distances to the person posing the expression. Because this study is the first of its kind, the results of this study offer only preliminary evidence that should not be generalized. That said, the results of the present study are as follows.

First and foremost, emotional expressions appear to influence the perceiver’s estimation of distance to the face of the expresser, particularly when they depict threatening or safe emotions. To a certain extent, this finding extends Teachman et al.’s (2008) demonstration of the emotional influence (fear of heights) on distance perception. The two threatening facial expressions differ because anger is more likely to signal approach or aggression by its expresser, but hate is more likely to signal withdrawal. However, both “safe” expressions (joy and pleasure) are more likely to signal a desire to approach the observer. Distances to these four opposing expressions were underestimated, indicating that participants perceived them to be closer than they actually were. With the exception of the 2 m condition, in which all facial emotions elicited distance underestimation, the two neutral expressions did not induce biased judgments. Not all emotions are associated with distinct behavioral tendencies (Seidel et al., 2010). For example, the valence associated with surprise, one of the two neutral emotions employed in this study, may vary depending on the context, that is, it may be positive if something occurs unexpectedly but negative if the expected outcome does not occur (Tops and Boksem, 2012), thereby triggering different actions. In the present study, however, these six emotions were chosen along the threat/safety dimension, with surprise and shame representing neutral valences. This may be why these two neutral emotions were more resistant to biased judgments.

However, distance interacted with facial emotion. Visual inspection of Figure 2 reveals that, for the safe and threatening expressions, the amount of underestimation grew with increases in distance, but for the two neutral expressions the amount of underestimation was greatest at 2 m. In distance perception literature (Baird and Biersdorf, 1967; Johnston, 1991; Norman et al., 1996), underestimation generally increases with distance. The rationale for the current result is not clear.

Of particular interest is the finding that the contrasting influences exerted by safe and threat vs. neutral expressions on distance perception were observed only up to 2 m, beyond which they disappeared. It appears that these four emotional expressions become more salient when the expresser is perceived as intruding on the observer’s personal space. More corroborating evidence is needed to confirm this finding.

Although female participants exhibited more bias in distance judgments (Figure 3), that does not mean that facial emotion exerted little influence on male participants. Perceptual effects of facial emotion across the three distance conditions were not equal, as indicated by the distance by emotion interaction (Figure 2). Absence of a three-way interaction involving participant gender suggests that facial emotions exerted similar influences on both male and female participants across the three distance conditions. Nevertheless, the finding that female participants were primarily responsible for the observed perceptual biases, particularly by the two safe and two threatening emotions, is consistent with the findings in the literature demonstrating women’s superiority in emotional competence overall (Eisenberg and Lennon, 1983; Barrett et al., 2000; Kret and De Gelder, 2012), but particularly in their capacity to recognize facial expressions of emotion (Hall, 1978; Hall et al., 2000; Thayer and Johnson, 2000; Hampson et al., 2006). It should be emphasized, however, that in the present study women’s superiority was reflected in their biased estimates of distance, particularly underestimating safe and threatening emotional expressions. It remains to be seen whether this bias is beneficial or not.

Unlike previous studies (Rotteveel and Phaf, 2004; Marsh et al., 2005; Seidel et al., 2010), the effect of actor gender was negligible in the present study. Perhaps one actor of each gender may not be sufficient to induce enough variance to reach statistical significance.

To conclude, the present study demonstrated that facial expressions of emotion (particularly threatening or safe emotions) influence others’ (predominantly female) judgments of how far away they are—but only within close proximity. It should be remembered, however, that this is the first study directed at perceptual effects of behavioral intentions conveyed by others’ facial expressions of emotion on estimating distances to them. Thus, until more corroborating evidence is found, caution should be exercised not to overgeneralize the present findings.

### Conflict of Interest Statement

The authors declare that the research was conducted in the absence of any commercial or financial relationships that could be construed as a potential conflict of interest.
